# Imaging Axonal Degeneration and Repair in Preclinical Animal Models of Multiple Sclerosis

**DOI:** 10.3389/fimmu.2016.00189

**Published:** 2016-05-19

**Authors:** Soumya S. Yandamuri, Thomas E. Lane

**Affiliations:** ^1^Department of Bioengineering, University of Utah, Salt Lake City, UT, USA; ^2^Department of Pathology, School of Medicine, University of Utah, Salt Lake City, UT, USA

**Keywords:** two-photon microscopy, axonal damage, remyelination, multiple sclerosis, animal models

## Abstract

Multiple sclerosis (MS) is a central nervous system (CNS) disease characterized by chronic neuroinflammation, demyelination, and axonal damage. Infiltration of activated lymphocytes and myeloid cells are thought to be primarily responsible for white matter damage and axonopathy. Over time, this neurologic damage manifests clinically as debilitating motor and cognitive symptoms. Existing MS therapies focus on symptom relief and delay of disease progression through reduction of neuroinflammation. However, long-term strategies to remyelinate, protect, or regenerate axons have remained elusive, posing a challenge to treating progressive forms of MS. Preclinical mouse models and techniques, such as immunohistochemistry, flow cytometry, and genomic and proteomic analysis have provided advances in our understanding of discrete time-points of pathology following disease induction. More recently, *in vivo* and *in situ* two-photon (2P) microscopy has made it possible to visualize continuous real-time cellular behavior and structural changes occurring within the CNS during neuropathology. Research utilizing 2P imaging to study axonopathy in neuroinflammatory demyelinating disease has focused on five areas: (1) axonal morphologic changes, (2) organelle transport and health, (3) relationship to inflammation, (4) neuronal excitotoxicity, and (5) regenerative therapies. 2P imaging may also be used to identify novel therapeutic targets via identification and clarification of dynamic cellular and molecular mechanisms of axonal regeneration and remyelination. Here, we review tools that have made 2P accessible for imaging neuropathologies and advances in our understanding of axonal degeneration and repair in preclinical models of demyelinating diseases.

## Introduction

Multiple sclerosis (MS) is a neuroinflammatory demyelinating disease affecting over 2.3 million individuals worldwide (1). Macrophages and T lymphocytes infiltrate the central nervous system (CNS), and along with resident microglia, release proinflammatory cytokines, culminating in oligodendrocyte damage and death (2). The resulting multifocal white matter demyelinating lesions cause dampening of saltatory signal transduction in axons (2–5). This ultimately leads to a variety of insidious clinical symptoms, including motor, cognitive, and behavioral deficiencies, such as numbness or pain in extremities, loss of vision, and dementia. These symptoms may manifest as episodes separated by functional recovery, termed relapsing-remitting MS, or may continue to progress with increasing debilities, termed progressive MS (6). It is believed that remyelination, reduced neuroinflammation, and adaptive CNS responses are responsible for remitting phases of disease (7–10). Therefore, in an attempt to prolong remission and prevent new lesion formation, disease-modifying therapies focus on reducing leukocyte trafficking or proliferation in the CNS. However, these FDA-approved therapeutics have only been indicated for relapsing-remitting MS. With 15% of MS patients diagnosed with progressive MS at onset (termed primary-progressive MS) and 80% of patients developing progressive MS within 20 years of diagnosis (secondary progressive), treatments for progressive MS have been long sought after yet remain elusive (11).

The historical focus on neuroinflammation and demyelination in MS may have delayed the development of effective treatments for progressive MS. Irreversible axonal damage and neurodegeneration have been identified as causative factors for chronic functional disability and progressive disease (11, 12). Indeed, patients with progressive forms of MS have increasing CNS atrophy in the spinal cord, cerebellum, and cerebral cortex, attributed to axonal loss (13–15). Progressive patients are also more likely to have decreased levels of *N*-acetyl aspartate within the brain, indicating a precipitous drop in neurons and corresponding evidence has shown an increase in neuronal apoptosis (12, 16, 17). Morphological indicators of axonal pathologies, such as ovoids, swelling, thinning, and transection are found in CNS biopsies of MS patients and are also evident in other neurodegenerative diseases (17, 18). Furthermore, internal structural changes have been found by immunohistochemistry. SMI32 is a monoclonal antibody that recognizes non-phosphorylated neurofilament, which is found in damaged axons. Accumulation of amyloid precursor protein (APP) is found in damaged axons due to transport deficiencies. Both SMI32 and APP positive axons have been found in demyelinating lesions in post-mortem MS biopsies (17–19). Importantly, the loss in neurons in progressive MS is attributed to axonal degeneration (20).

**Figure 1:**
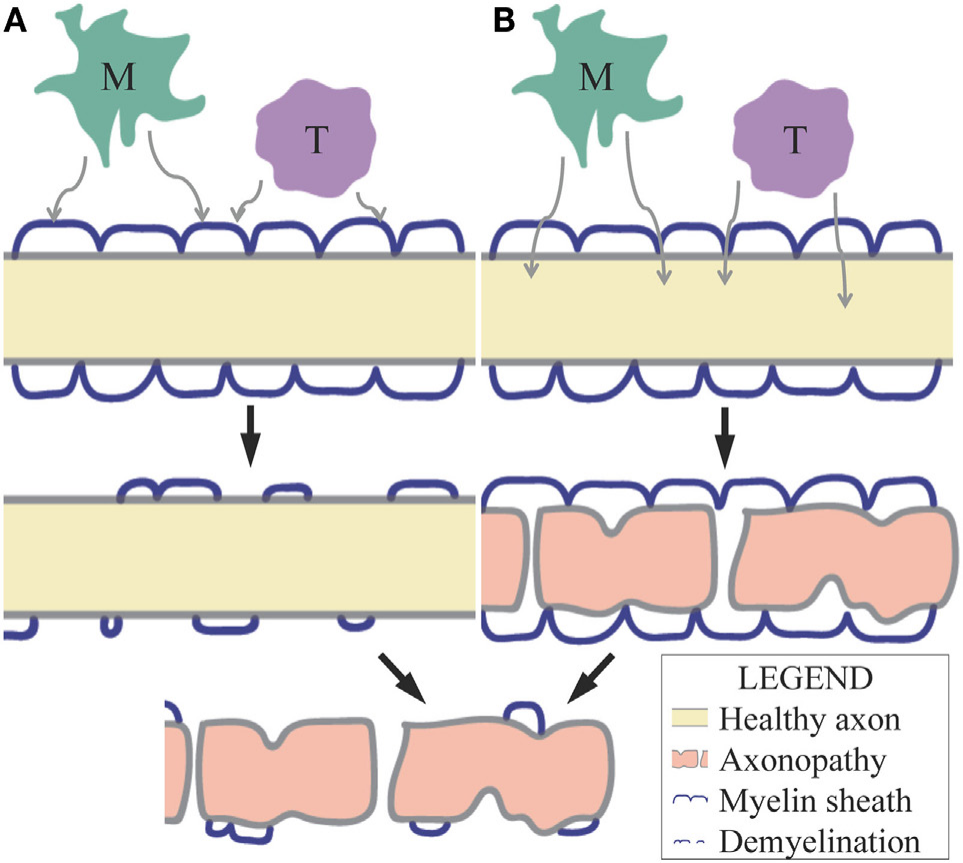
**(A)** The outside-in model of axonal degeneration predicts that demyelination occurs before axonopathy due to myelin-degenerative factors released by inflammatory cells (M = macrophages/microglia, T = T lymphocytes) within the CNS. The loss in protection and trophic support by myelin results in axonal damage in this model. **(B)** The inside-out model of axonal degeneration or Wallerian degeneration predicts that axonopathy occurs before demyelination due to neurotoxic factors released by inflammatory cells within the CNS. These factors may diffuse through myelin or pass through the nodes of Ranvier.

Causation of MS axonopathy has been contested. Inflammatory demyelinating lesions can be identified in patients by magnetic resonance imaging (MRI) or in post-mortem CNS biopsies by Luxol fast blue (LFB) staining. Historically, axonal loss has been considered a result of demyelination. This is the “outside-in-model” of axonal degeneration in demyelinating disease (Figure 1A). More recently, axon loss has also been found in non-lesional white matter of post-mortem MS biopsies, as identified by MRI or LFB, termed “normal appearing white matter” (NAWM) and indicating axonal damage in the absence of inflammation and demyelination (21). This form of axon damage is referred to as Wallerian degeneration or the “inside-out-model” of axon degeneration, where the myelin sheath has the appearance of an empty tube, due to axon loss within the myelin sheath (Figure 1B). The relative importance of these two models is significant in understanding optimal treatment strategies: should axon and neurodegeneration be targeted for treatment or is prevention of demyelination sufficient to save axons and neurons?

Many factors contribute to axonal degeneration, complicating our ability to confirm whether demyelination or axonopathy occurs first. The lack of physical and trophic support from the myelin sheath may contribute to axonal loss (22, 23). However, axonal damage is not always secondary to demyelination. Axonopathy has also been attributed to attack by inflammatory cells and their secretion of inflammatory cytokines and toxic species (22–25). Increased macrophage and microglia density within the CNS microenvironment have been attributed to axonal loss (17, 24). Further investigation showed that microglia contact and surround axons and dendrites in cortical lesions (17). Some microglia encapsulate the transected ends of axons in white matter lesions, implying a direct involvement in degenerating axons. In addition, CD8^+^ T cell density is also correlative with axonal damage in MS patient biopsies (24).

Myeloid cells may also cause axonal damage by releasing neurodegenerative molecules, such as glutamate, reactive oxygen species (ROS), and reactive nitrogen species (RNS) (26–28). Furthermore, astrocytes and oligodendrocytes have decreased expression of glutamate clearance and metabolism receptors in MS lesions (28–30). As a result, increased glutamate has been detected in the cerebrospinal fluid of MS patients and within tumefactive demyelinating lesions (31, 32). An increase in glutamate receptors on neurons increases their vulnerability to excitotoxicity and degeneration (28–30). ROS, RNS, and glutamate may also traverse the myelin sheath by diffusion or through the nodes of Ranvier, degenerating the axon, and highlighting one possible cause of Wallerian degeneration in myelinated axons (33–35).

Activation of neuronal glutamate receptors by glutamate and RNS upregulates extracellular ROS and RNS release and intracellular [Ca^2+^] (36–39). Increased intracellular [Ca^2+^] activates neurofilament fragmenting proteases, which may cause the transport deficiencies seen in degenerated axons (40). Intracellular [Na^+^] also increases due to an increase in axonal Na^+^ channels, a neuronal adaptive response to demyelination, and due to increased import of Na^+^ by Na^+^/Ca^2+^ exchangers that expel excess intracellular Ca^2+^ (33, 41, 42). As a result, Na^+^/K^+^-ATPase activity rises to expel excess Na^+^, causing depletion of ATP (43–48). Energy deficiencies are exacerbated by mitochondrial functional impairment caused by ROS and RNS, as well as insufficient mitochondria along the length of the axon due to the previously mentioned axonal transport deficiencies (43–48). Therefore, ROS, RNS, and glutamate cause impairment of neurofilament integrity, mitochondria density and function, energy metabolism, and ATP synthesis in axons (28, 31, 49–52). As is the case in any biological system, a large number of factors play a role in axonal degeneration, with varied relationships, feedback loops, and redundancies: inflammation causes demyelination, demyelination causes axonopathy, inflammation causes axonopathy, and demyelination enhances inflammation. These interconnected relationships increase the difficulty of finding a single therapeutic target for progressive MS.

Histopathology, confocal imaging, and molecular biology techniques have yielded insight into some of these relationships; however, a definitive understanding of axonal degeneration in MS is still lacking. The mechanisms that result in neurodegeneration are both complex and dynamic, with axon damage caused by inflammatory cell attack, inflammatory cytokines, excitotoxic molecules, and myelin damage (22–25). This process also involves flux in molecular and ion concentrations, morphologic changes, internal structure and organelle damage, and electrical conductivity dysfunction. The highly dynamic and multi-variate nature of axonal degeneration makes it a well-suited beneficiary of real-time imaging. In the last half-decade, the convergence of 2P microscopy, preclinical murine MS models, and various transgenic animals and dyes have allowed for real-time visualization of axonal degeneration with respect to morphologic changes, organelle transport and damage, inflammation and axon health, neuronal excitotoxicity, and regenerative therapeutics.

## The Tools

### Preclinical Animal Models of MS

Thus far, 2P microscopy has been used to study axonal degeneration following infection of mice with the neurotropic JHM strain of mouse hepatitis virus (JHMV) or induction of experimental autoimmune encephalomyelitis (EAE) (53–57). While these models are independent and unique from one another, they both share important histologic features with MS, including neuroinflammation, demyelination, and neurodegeneration. EAE is the most commonly used preclinical murine model of MS. By the mid-1900s, researchers discovered that injection of emulsified CNS tissue or transfer of lymph node cells from animals sensitized with CNS tissue induced encephalomyelitis in a variety of animal hosts (58–61). Today, “active” EAE is initiated by injection of defined encephalitogenic myelin protein epitopes in combination with complete Freund’s adjuvant (CFA), whereas “passive” EAE is induced by transfer of encephalitogenic myelin-sensitized T lymphocytes (60, 62–69). Some of these EAE models also require administration of the microbial-based immunologic adjuvant pertussis toxin (PT), which is thought to enhance EAE induction by increasing blood–brain barrier (BBB) permeability, expanding myeloid cells and antigen-specific T lymphocytes, reducing regulatory T lymphocytes, and modulating expression of inflammatory cytokines (70–72). PT is often administered in donor mice for passive EAE and in active EAE mice, depending on the murine strain and immunizing antigen (73). Like in MS, the EAE clinical course can be highly variable; researchers can achieve an intended MS-like clinical course based on murine strain, injected antigen, and adjuvant (74).

All active EAE 2P studies of axonopathy have been induced by injection of C57BL/6 mice with myelin oligodendrocyte glycoprotein (MOG) peptide fragment 35–55 (MOG_35–55_) in conjunction with CFA, stimulating a robust T cell response. In these studies, PT is injected on days 0 and 2 post-immunization, resulting in neuroinflammation and disease induction. Axonal density in MOG_35–55_ immunized C57BL/6 mice is significantly decreased in lesions, NAWM, and gray matter by disease onset, when measured by Bielschowsky Silver impregnation (75). SMI32 and APP positive axons are found prior to disease onset and demyelination (76). Interestingly, T cell infiltration is very low at this point; rather, astrocyte hypertrophy is widely observed concomitant with axon injury (76). These data demonstrate that axonal damage may precede detectable demyelination in EAE.

A number of viral pathogens have been implicated in human MS, including Epstein–Barr virus (EBV) and human herpesvirus-6 (HHV-6) (77, 78). The differential prevalence of MS by geographic location and the increased risk to individuals who relocate to high prevalence geographies may be attributed to a viral infection (79). Therefore, preclinical viral models of MS are important tools for understanding disease pathogenesis and testing potential therapeutic mechanisms. JHMV is a positive-sense single-stranded RNA virus of the *Coronaviridae* family. Intracranial injection of JHMV into C57BL/6 mice results in acute encephalomyelitis and chronic inflammatory demyelination and axonopathy in the CNS (80). While JHMV infects and replicates within oligodendrocytes, astrocytes, and microglia, demyelination occurs due to T lymphocyte-directed responses against viral antigens and macrophage-mediated stripping of myelin, and not virus-induced apoptosis/necrosis of oligodendroglia (81, 82). Clinical symptoms include ataxia and lower limb paralysis that start 1 week after infection and peak 2–3 weeks later. After peak disease, low-level inflammation and demyelinating lesions are present, and this is likely due to viral persistence below the level of detection.

Studies using immunodeficient RAG1^−/−^ mice (lacking functional T and B lymphocytes) have indicated that CD4^+^ and CD8^+^ T lymphocytes as well as macrophages are key contributors to demyelination in JHMV-infected mice (82, 83). However, JHMV-infected RAG1^−/−^ mice exhibit a significant increase in SMI32 immunolabeling in comparison to naïve mice despite a lack of demyelination (83). SMI32 reactivity coincides with areas of macrophage and microglial infiltration and activation. There is a further significant increase in SMI32 immunolabeling when JHMV-infected RAG1^−/−^ mice receive splenocytes from JHMV-immunized wild-type C57Bl/6 mice, though there was no statistical difference after adoptive transfer with splenocytes depleted of CD4^+^ and CD8^+^ T cells. These data suggest that demyelination is not necessary for axonal damage to occur in JHMV-infected mice in that damage may be mediated by factors that can traverse the myelin sheath, but T cell inflammation and demyelination significantly exacerbate axon damage. Thus, both the inside-out and outside-in models of axon degeneration are perpetuated in JHMV according to these studies (82, 83).

Local administration of glutamate or nitric oxide donors induce axonopathy in mice and have also been used to understand mechanisms of axonal degeneration and regeneration in the context of demyelinating disease (57, 84). Thus, autoimmune, viral, and excitoxicity-inducing models have all provided clues into how axonal damage may precede demyelination. However, an understanding of real-time interactions and visualization of cause-and-effect cannot be fully realized in static snapshots. While histopathology, flow cytometry, and molecular biology techniques have provided important data regarding axon biology in the CNS during demyelinating disease, this has largely focused on markers of axon damage and the presence of various inflammatory cells and glia. We, along with others, have employed 2P imaging to better understand the dynamic changes in axonal morphology and pathology in models of neuroinflammation, excitotoxicity, and cell replacement therapies.

### 2P Imaging and Setup for Murine MS Models

In confocal microscopy, a single photon excites a fluorescent molecule to a higher energy state. In 2P imaging, two lower energy photons simultaneously excite a fluorescent molecule to a higher energy state. In order to achieve a high enough photon density for simultaneous absorption, spatial and temporal restriction is imposed. A high power laser is focused in pulses: during the peak of these pulses, there is enough energy to create 2P excitation. In order to create an image, the focused laser scans over the target tissue and the spatial photon density drops rapidly above and below the focus, preventing out-of-focus excitation, ultimately preventing background and photobleaching of surrounding areas. Therefore, background signal is significantly decreased in 2P microscopy compared to one-photon confocal microscopy. Furthermore, the longer excitation wavelength results in decreased scattering, auto-fluorescence, and phototoxicity. Due to this decreased scattering, 2P microscopy ideally allows imaging up to 1 mm in depth, while confocal microscopy is limited to 200 μm. As a result, this technology may be utilized to visualize thicker tissue sections, tissues *in vivo*, and whole organs *in situ*. Therefore, 2P imaging provides the ability to monitor cellular and molecular movement, velocity, location, and interactions within the endogenous CNS.

2P microscopy has been used to image axons in the brainstem, dorsal spinal cord, and ventral spinal cord of murine MS models. While the brainstem and dorsal spinal cord may be accessed *in vivo*, due to limited imaging depth, it is necessary to extract the spinal cord and image it *in situ* to capture dynamics in the ventral region. Therefore, in order to capture exogenously engrafted GFP-expressing neural precursor cell (NPC) dynamics in the ventral spinal cord, Greenberg et al. (53) developed a novel technique to extract and image the spinal cord *in situ*. After sacrifice (omitting cervical dislocation), a full laminectomy is performed from cervical vertebra 1 through the lumbar spinal column. The spinal cord is carefully resected at the necessary levels and removed. The spinal cords are then embedded in agarose to maintain stability during imaging and perfusion with warmed, oxygenated media.

Standard setup in *in vivo* imaging of the brainstem or spinal cords involves anesthetization, maintenance of body temperature, tracheotomy or intubation, maintenance of respiration, and circulation of saline solution or mouse artificial cerebrospinal fluid at the exposed imaging site. In order to expose the brainstem, it is necessary to incline the head to enhance imaging depth and remove the musculature and dura mater between cervical vertebra 1 and the base of the skull. A sterile agarose patch must be kept here to dampen breathing and heartbeat artifacts. This technique allows visualization of the dorsal medulla oblongata, caudal cerebellum, and upper spinal cord, and has been adapted from Gobel and Helmchen (85). In order to visualize the dorsal spinal cord, it is necessary to perform a laminectomy and remove the dura mater at the appropriate spinal cord levels, a technique first described by Kerschensteiner et al. (81) in 2005.

### Transgenic Animal Lines

Transgenic murine lines have facilitated the use of 2P to study axonal degeneration in preclinical MS models. Morphology, location, motility, concentration, and interactions of endogenously labeled cells, organelles, and/or various molecules can be monitored during different disease states. One of the most widely used transgenic animal line for the study of axonopathy in preclinical MS models is the Thy1-XFP mouse. Thy1-XFP mice express a spectral variant of GFP within the cytoplasm of a subset of neurons (86). Axonopathy in EAE and JHMV is most commonly studied in the brainstem or spinal cord of Thy1-YFP or Thy1-GFP mice, where a large number of medium-to-large caliber axons fluoresce YFP or GFP, respectively. Thy1-CFP mice have been used to a lesser extent, due to sparser axonal fluorescence. Thy1-XFP mice have been crossed with CD2-GFP^20^ or Cx3cr1^GFP/+^ mice to visualize T lymphocytes or macrophages/microglia, respectively, and their interactions with axons in active EAE (54).

Thy1-XFP mice have also been crossed with Thy1-MitoCFP or Thy1-PeroxiYFP mice, in order to visualize mitochondria or peroxisomes, respectively, within axons (54, 57). Thy1-EB3-YFP mice have been developed to investigate the axonal transport changes that occur during degeneration. Microtubules maintain axonal structure and directional transport within axons. End-binding protein 3 (EB3) is one of three microtubule plus-end tracking proteins that facilitate microtubule growth at their growing ends (87). EB3 expresses YFP in a subset of axons in Thy1-YFP-EB3 mice. These fluorescent EB3s resemble comets, and the speed, directional orientation, and length of these comet-like EB3 structures can be monitored as an indicator of microtubule outgrowth changes during pathology (57).

Recently, Förster resonance energy transfer (FRET)-based genetically encoded Ca^2+^ sensors (GECIs) have been used to measure [Ca^2+^] within neurons during pathology in preclinical MS models (55, 56). Thy1-TN-XXL and Thy1-CerTN-L15 transgenic mice express Troponin C, bound to CFP on one end and YFP on the other (88, 89) Troponin C is a Ca^2+^ binding protein that exhibits an expanded conformation during baseline levels of intracellular [Ca^2+^]. In this state, excitation at the absorbance wavelength of CFP results in CFP emission. Upon an increase in intracellular [Ca^2+^], Troponin C binds Ca^2+^ and contracts, bringing CFP and YFP closer to each other. During excitation at the CFP absorbance wavelength, energy transfers from CFP to YFP due to their proximity, and YFP signal is emitted. Thus, changes in axonal [Ca^2+^] can be monitored by calculating the ratio of CFP and YFP.

### Cellular and Molecular Labeling

A variety of techniques enable visualization of cellular and molecular activity in murine MS models aside from the use of transgenic animals. Tissues can be stained *in vivo*, fluorescent cells can be transplanted from transgenic animals, and cells can be labeled *in vitro* and subsequently transplanted. The former two techniques have been utilized in the study of axonal pathology in murine MS models. Romanelli et al. (86) published a protocol for staining spinal cord tissue *in vivo* for 2P imaging. This protocol has been used to label myelin using FluoroMyelin stain (Invitrogen), Cell Trace BODIPY TR methyl ester dye (Invitrogen), or Nile red stain and inflammatory cellular infiltrates in Thy1-XFP animals (54, 56). Vital dyes have also been used with 2P imaging to visualize real-time changes in relevant molecular concentrations or signals, such as hydrogen peroxide (ROS), measured with AMPLEX UltraRed reagent (Invitrogen); nitric oxide (RNS), measured with DAF-FM diacetate reagent (Invitrogen); and mitochondrial membrane potential, measured with tetramethylrhodhamine (TMRM), methyl ester (Invitrogen) (54).

Siffrin et al. (56) created bone marrow chimeric mice in order to visualize the activity of RFP-expressing bone marrow-derived peripheral immune cells (CD45^+^) in Thy1-EGFP (tdRFP → Thy1-EGFP) animals in which EAE was induced. This group also initiated EAE in Rag1^−/−^ × Thy1-EGFP mice by injection of *in vitro* differentiated MOG_35–55_-specific TCR transgenic (2d2) Th17 cells from tdRFP mice (2d2.tdRFP Th17). T helper cells were isolated from total splenocytes using CD4^+^CD62L^+^ magnetic bead cell sorting. These cells were then stimulated with MOG peptide and cytokines to induce specificity and differentiation into Th17 cells. These cells were also transplanted into RAG1^−/−^ × Thy1-EGFP animals following induction of EAE via injection of non-fluorescent 2d2 Th17 cells, allowing visualization of RFP-expressing Th17 cells in relation to EGFP axons. IL-17A-enriched 2d2.tdRFP Th17, 2d2.tdRFP Th1, and ovalbumin-specific TCR transgenic Th17 (OT-2.tdRFP Th17) cells, which were derived in a similar manner *in vitro*, were also visualized after transplantation into non-fluorescent 2d2 Th17-induced EAE RAG1^−/−^ × Thy1-EGFP animals. As a result, it was possible to monitor dynamics and interactions of Th1 and Th17 cells with varied differentiation status and specificity in relation to EAE-induced axonopathy.

Our laboratory has used NPCs derived from GFP (GFP–NPCs) and PLP–GFP (PLP–GFP–NPCs) mice; these cells express GFP constitutively or under control of the proteolipid protein (PLP) promoter, respectively (53). Through the use of 2P microscopy to monitor live interactions, we verified previous histologic evidence of NPC differentiation upon interaction with damaged axons in JHMV-infected Thy1-YFP mice. Thus, a large variety of experiments can be conducted with selected transgenic mouse lines in combination with 2P imaging. With the addition of vital dyes, which do not require the time and expense of animal care, the number of variables that can be simultaneously monitored dramatically expands.

### Technical Considerations

Some technical limitations must be considered when using 2P microscopy. Photobleaching must be mitigated by using a low laser power. Compensatory equations, such as a monoexponential decay adjustment have been utilized to normalize for this phenomenon (55). Variance in scattering by different fluorophores may give false impression of relative emission intensity with depth. Ratiometric signaling constructs, such as those used in GECIs, may be affected by this. Since CFP scatters more than YFP, the YFP/CFP ratio of axons within the brainstems of Thy1.CerTN-L15 or Thy1-TN-XXL mice will increase with depth, giving a false impression of slight [Ca^2+^] increase (55, 56). A linear correction may be useful in compensating for the differential scattering.

Considering the depth limitation of 2P microscopy, alternative methods may aid in gaining a complete understanding of pathology in the CNS. MRI may be utilized to identify deep lesions of inflammation, axonal damage, and demyelination in live mice (90–92). Following lesion identification, it may be possible to take tissue sections of deep lesions and visualize live cellular and molecular dynamics *in situ* using 2P microscopy (93). Alternatively, a variety of techniques have been developed in order to make tissue sections and whole organs transparent (94–96). Transparentization reduces scattering and allows greater imaging depth with both 2P and confocal microscopy. However, this process requires fixation; thus, it can only be used to visualize a static snapshot of pathology.

Vital dyes may exhibit non-specific binding, compartmentalization, and leakage (97). Therefore, it is necessary to minimize variations in preparation of dye, location of dye administration, dye incubation time, imaging duration, and location of imaging. While transgenic animal lines may seem superior in consideration of these limitations, the additional data that can be easily gained with these dyes makes them an irreplaceable boon to live imaging at this current time.

Given the variety and advancement of techniques to visualize cells, organelles, and various molecules, one must consider the limitation posed by overlap of emission spectra. Generally, a limited number of channels can be detected at a time. Furthermore, while many transgenic variants and vital dyes have been made, their emission spectra are most often GFP or RFP, and to a lesser extent YFP or CFP have been used. There must be enough variety such that each variable in an experiment has a different emission spectrum.

## Advances in Understanding Axonal Degeneration and Repair

### Axon Morphologic Changes

Using time-lapse 2P imaging to look at axons in Thy1-YFP mice in the EAE and JHMV models, a novel temporal connection was made between the axonal swelling, ovoids, transections, and thinning that had been described in MS and other neurodegenerative diseases (53, 54). In 2011, Nikic et al. (54) described the reversible and progressive stages of axonal pathologies observed with 2P microscopy in EAE mice post-onset, termed focal axonal degeneration (FAD). FAD consists of three stages: stage 0 is a normal appearing axon, a stage 1 axons has focal swellings, and a stage 2 axon has transections. Axon pathology can progress or reverse through these stages. Stage 1 axons may progress to stage 2 or regress to stage 0. Importantly, stage 2 axons were not observed to revert back to stage 0.

**Figure 2:**
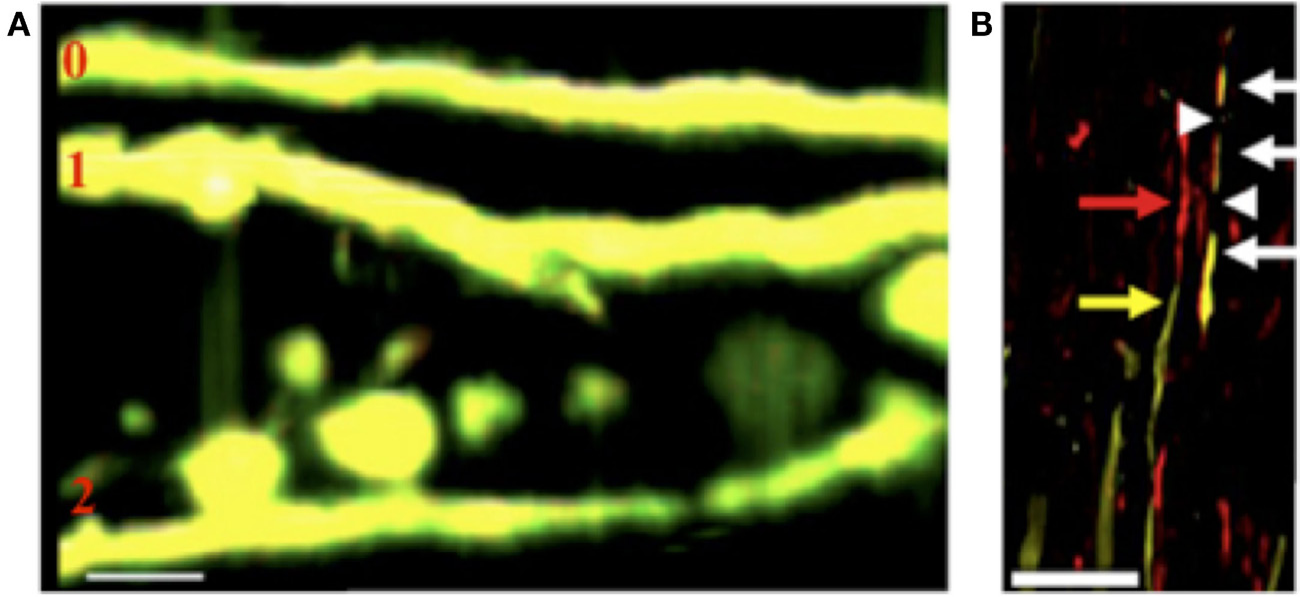
**(A)** Representative 2P image of axons in JHMV-infected Thy1-YFP mice shows FAD stages 0, 1, and 2, 21 days post-infection (53). **(B)** Representative image of SMI32 immunohistochemistry (non-phosphorylated neurofilament) in Thy1-YFP mice, 23 days post-infection shows YFP^+^SMI32^−^ sections (yellow arrow), YFP^+^SMI32^+^ sections (white arrow), YFP^−^SMI32^+^ sections (red arrow), and YFP^−^SMI32^−^ sections (white arrowhead) (53). (Scale bar = 20 μm).

Using a 2P approach, we observed that FAD also occurs in JHMV-infected Thy1-YFP mice early following CNS infection (Figure 2A) (53). Upon further immunolabeling with SMI32, we detected additional nuances to the FAD pathologies previously described. Axons could have any combination of YFP^+^SMI32^−^, YFP^−^SMI32^+^, YFP^+^SMI32^+^, and/or YFP^−^SMI32^−^ sections: this means that parts of axons could be considered healthy and intact, intact but damaged, or transected (Figure 2B). The detection of YFP^−^SMI32^+^ axons indicated that YFP loss may not be sufficient to indicate complete transection. Subsequent immunolabeling with SMI31 to detect “healthy” phosphorylated neurofilament showed that SMI31 immunoreactivity was exclusive to YFP^+^ axons.

These data also contributed to a historically important question regarding axonal degeneration in demyelinating disease: all three stages were detected in myelinated axons, providing traction for the inside-out model of axonal degeneration. FAD 1 and 2 axons were found in fully myelinated axons, though there were more partially or fully demyelinated stage 1 and stage 2 axons (54). This corroborates previous MS data in that axonal degeneration can occur in myelinated axons, but is more prevalent in demyelinated axons. Inflammation has been ubiquitously linked to axonal degeneration. Using time-lapse 2P imaging, Nikic et al. (54) also found that stage 1 axons were more likely to degenerate into stage 2 axons during peak clinical symptoms and elevated inflammation, while recovery was more likely even 1 day after peak symptoms. Using 2P imaging to complement standard laboratory techniques and transgenic animals, our understanding of the temporal characteristics of axonal degeneration in demyelinating disease has been corroborated and advanced.

### Organelle Transport and Damage

Morphologic changes in axons are accompanied by organelle damage and transport deficiencies (98). Though normally undetectable by immunolabeling, APP is detected when it accumulates due to defective axonal transport. It has been found in acute lesions and at the borders of active chronic MS lesions (19). 2P imaging of spinal cords of Thy1-EB3-YFP EAE mice show that FAD 1 axons have a significant increase in EB3 “comets” oriented in the retrograde direction compared to FAD 0 axons or axons in control mice (57). Microtubule orientation is also highly variant. EB3 is most often parallel to the axon; however, upon induction of EAE, FAD1 axons exhibited comets oriented in various angles.

In order to continue to understand transport deficiencies, Sorbara et al. (57) visualized axonal mitochondria and peroxisomes. 2P microscopy has allowed for visualization and quantification of velocity, directionality, location, and stop duration. In concordance with the EB3 data, both anterograde and retrograde mitochondrial flux is significantly decreased in FAD1 axons (57). Furthermore, both mitochondrial and peroxisomal flux is significantly decreased in FAD 0 EAE axons compared to control axons, indicating that transport deficits may occur before morphological changes to axons. Mitochondrial speed is significantly decreased, stop duration is significantly increased, and the frequency of stops is significantly increased in the anterograde direction in EAE axons. As a result of these mitochondrial transport deficits, a significant reduction was seen in organelle density in distal portions of axons, matching the increase in retrograde-oriented EB3. The lack of organelles, such as mitochondria, throughout the length of axons may have significant impact on maintaining neuronal homeostasis and health.

Tracking mitochondria to monitor transport deficiencies in MS models is highly useful, considering that it has also been identified as an early marker of axonal pathologies. Cortical biopsies of MS patients have shown decreased mRNA expression and impaired activity of mitochondrial electron transport chain complexes (50–52). These effects may be correlated with inflammation in that a decrease in axonal mitochondria complex IV activity was associated with an increase in local macrophage/microglial density (52). These findings corroborate the complex interaction of inflammation, axonal health, and mitochondrial health and density.

Deficient transport of mitochondria throughout axons is complemented by mitochondrial structural and functional changes. Parallel to evidence that mitochondria transport deficits occur in “normal” FAD 0 axons, mitochondrial dysfunction and dysmorphia precede morphologic changes of axons (54). Electron microscopy revealed swollen, rounded mitochondria within FAD 1 axons of EAE-induced mice. Given this information, EAE was induced in Thy1-YFP-16 × Thy1-MitoCFP mice for visualization of mitochondrial morphology and membrane potential with 2P imaging. Quantification of these factors revealed that decreased shape factor (indicating dysmorphia) correlated with abnormally low mitochondrial membrane potential. While dysmorphic mitochondria were not found in either control animals or MOG_35–55_ immunized animals prior to disease onset, swollen mitochondria were found in FAD 0 axons located within inflammatory lesions. In concordance with previous histologic MS data, axonal mitochondria damage was localized to areas of increased macrophage and microglial inflammation, perhaps due to release of ROS and RNS (17, 25, 49–52).

Reactive oxygen species and RNS were significantly increased in lesions of EAE mice upon onset (54). Application of these species to healthy Thy1-YFP-16 × Thy1-MitoCFP mice spinal cord showed that both result in mitochondrial and FAD changes, similar to what occurs in EAE, but without demyelination. Examination of active lesions from MS biopsies showed mitochondrial damage regardless of demyelination state, similar to data showing fully myelinated FAD 1 and 2 axons (54). Therefore, 2P imaging provides significant evidence that axonal damage and related transport and mitochondrial deficiencies occur in areas of inflammation, and release of ROS and RNS species by macrophages and microglia may be one contributor of axonal damage.

### Inflammation and Axon Health

Inflammatory cells have been found to contact axons and cause degeneration in demyelinating disease. SMI32 reactive axons were found in areas with high macrophage and microglial density in JHMV spinal cord (83). In MS, APP reactive axons increased in a correlative manner with CD8^+^ T lymphocyte and macrophage/microglia density in lesions and periplaque white matter (24). Furthermore, the pattern of axonal transections in active and chronic active lesions matched that of MHC class II-positive macrophages and microglia (18). Apposition, contact, and engulfment of axons, dendrites, and soma by microglia have been detected in MS active and chronic active cortical lesions, and correlate with axonal damage and transection (17). Using 2P microscopy, Nikic et al. (54) also found an increase in macrophage and microglial density local to axons in acute EAE lesions in Cx3cr1^GFP/+^ × Thy1-CFP-S mice.

Siffrin et al. (56) used 2P microscopy in the brainstems of EAE mice to understand the motility characteristics of Th17 cells, which have been implicated in causing clinical and histologic disease upon induction of EAE. CD45^+^ cells in MOG_35–55_-induced active EAE in bone marrow chimera tdRFP → Thy1.EGFP mice and adoptively transferred *in vitro* differentiated 2d2.tdRFP Th17 cells for passive induction of EAE in RAG1^−/−^ × Thy1-EGFP mice both showed high motility and invasiveness into the parenchyma. However, 2d2.tdRFP Th17 cells showed decreased velocity during peak and chronic disease, while CD45^+^ cells maintained their velocity through all disease stages with the exception of remission. This decrease in velocity was associated with decreased displacement rate and increased meandering during disease. This corroborates the notion that antigen-specific T cells stop upon recognition of cognate antigen presented by MHC molecules as previously shown in EAE (99–101).

Consequently, Siffrin et al. (56) induced passive EAE in RAG1^−/−^ × Thy1-EGFP mice by adoptive transfer of non-fluorescent 2d2 Th17 cells to visualize the effects of differentiation status and antigen-specificity on interactive behavior. This study compared 2d2.tdRFP Th17, IL-17A enriched 2d2.tdRFP Th17, OT-2.tdRFP Th17, and 2d2.tdRFP Th1 cells. All three Th17 cell types had higher mean velocities and displacement rates than Th1 cells. Further analysis showed that the Th17 cells increasingly scanned dysmorphic axons in lesions: they contacted neurons more frequently and longer, particularly at peak disease. On the other hand, Th1 cells did not seem to undergo motility changes akin to antigen recognition, rather engaging in random, non-specific, and abbreviated contacts.

However, these data also indicate that Th17-neuronal contact is antigen independent as both 2d2.tdRFP and OT-2.tdRFP Th17 cells engaged in these neuronal interactions. Using electron microscopy and *in vitro* co-cultures with immunocytochemistry to further characterize these interactions, Siffrin et al. (56) found contacts between T lymphocytes and various myelinated and unmyelinated regions of neurons. At the point of contact, T lymphocytes exhibited polarization of organelles and microtubule-organizing centers, similar to immune synapses. These contacts were found to be MHC class II and antigen independent, but dependent on adhesion molecule LFA-1, which clusters at immune synapse contact sites and targets cytolytic granules to the cell surface (102). While Th17 cells caused neuronal death in co-cultures, Th0 and Th1 cells, and Th17 supernatants did not. These data imply that Th17 cells make an immune synapse-like contact with neurons, especially during peak disease, potentially causing neuronal degeneration and death. The discovery of this remarkable interaction was highly dependent on the ability to visualize these interactions in real time.

### Neuronal Excitotoxicity

Sustained Th17-neuronal contacts in EAE correlated with intracellular [Ca^2+^] increase, an early sign of axonal pathology (38, 103). Increased axon [Ca^2+^] was seen upon CD45^+^ cell contact, which decreased upon separation in MOG-EAE-induced bone marrow chimeric tdRFP→Thy1.Tn-XXL mice (55). Thy1.CernL15 animals induced with passive EAE by adoptive transfer of 2d2.tdRFP Th17 cells into RAG1^−/−^ × Thy1-CernL15 or induced with active MOG-EAE in tdRFP→Thy1.CernL15, showed localized [Ca^2+^] increase within contacting neuronal processes and soma in EAE lesions (56). The highest [Ca^2+^] increase in the Thy1.CernL15 model was found at the borders of active demyelinating lesions, which had a high amount of disintegrating myelin and debris. Sustained contact resulted in achievement of critical [Ca^2+^] signal, which preceded axon dissection at contact site and irreversible damage in soma. When quantifying GFP voxels as an indicator of neurodegeneration, decreased neuronal tissue was found in the presence of 2d2 Th17 cells; increased APP immunolabeling corroborated these results. Indeed, 2d2 Th17 caused a more progressive disease, with decreased remissions and increased mortality compared to 2d2 Th1 cells. Interestingly, injection of ovalbumin-specific CD4^+^ T cells resulted in reduced [Ca^2+^] elevation in axons. Though velocity and contacting dynamics were not found to be antigen specific, axonal [Ca^2+^] changes may be.

Glutamate excitotoxicity also causes an increase in axonal [Ca^2+^] (38, 39). Inhibition of NMDA glutamate receptors in EAE has shown decreased neurologic symptoms and inflammatory cytokine production (104–107). 2P imaging showed that local administration of glutamate to the brainstem of Thy1.TN-XXL mice resulted in local axonal [Ca^2+^] increases as well as morphologic changes, such as swelling and transections. These data indicate the variety of changes occurring in axons in preclinical MS models and the wide array of potential therapeutic targets, including glutamate excitotoxicity, morphologic changes, ion concentration changes, and inflammatory cell contact.

### Regenerative Therapeutics and Neural Precursor Cells

Drugs/molecular therapeutics and NPCs have been investigated for their potential to alleviate axonal pathologies in MS models. The molecular therapeutics tested are FDA-approved MS drugs, their biological byproducts, axonal channel/receptor blockers, and ROS/RNS scavengers. These therapeutics and their effects on axonal pathology as monitored by 2P microscopy are summarized in Table 1.

**Table 1:** **Molecular therapeutics for axonal regeneration and results of 2P imaging**.

Therapeutic	Disease model	2P imaging results	Reference
FTY720, FTY720-P, MMF, or DMF (MS drugs and analogs)	Glutamate delivery	Delayed [Ca^2+^] increase or decreased [Ca^2+^]; slower or decreased FAD	Luchtman et al. (84)
MK-801 (NMDAR blocker)	Glutamate delivery	Prevention of FAD	Luchtman et al. (84)
MK-801 (NMDAR blocker)	EAE	Intracellular [Ca^2+^] reduction to baseline in Th17 contacting axons	Siffrin et al. (56)
PHT (Na^+^ channel blocker)	EAE	Intracellular [Ca^2+^] reduction in Th17 contacting axons, less than MK-801	Siffrin et al. (56)
FeTPPs, PBN, and EUK134 (ROS and RNS scavenger cocktail)	EAE	Recovery of FAD 1 axons	Nikic et al. (54)
cPTIO (RNS scavenger)	Spermine (RNS donor) delivery	Rescue of mitochondrial velocity (anterograde and retrograde)	Sorbara et al. (57)
Methylprednisolone (Corticosteroid)	EAE	Rescue of mitochondrial velocity (anterograde and retrograde)	Sorbara et al. (57)
FeTPPs, PBN, and EUK134 (ROS and RNS scavenger cocktail)	EAE	Rescue of mitochondrial velocity (anterograde and retrograde)	Sorbara et al. (57)

*FTY720, Fingolimod; FTY720-P, FTY720-phophate; DMF, dimethyl fumarate; MMF, monomethyl fumarate; PHT, phenytoin; FeTTPS, 5,10,15,20-Tetrakis(4-sulfonatophenyl)porphyrinato Iron (III), chloride; PBN, N-tert-butyl-α-phenylnitrone; cPTIO, 2-4-carboxyphenyl-4,4,5,5-tetramethylimidazoline-1-oxyl-3-oxide*.

The allure of stem cells lies in their ability to differentiate and replace lost tissue as well as their release of regenerative factors. The therapeutic potential of neural stem cells (NSCs) and NPCs has been investigated in demyelinating disease, especially in regard to their ability to myelinate axons. Numerous transplantation studies in demyelinating models, such as EAE and shiverer mice, a genetic model of dysmyelination, have shown that NSC/NPC-based therapy decreases inflammation and increases the number of myelinating cells in the CNS (108–111). These therapies include bone marrow mesenchymal stem cell-derived NSCs, oligodendrocyte progenitor cells, and human CNS NSCs. Transplantation of allogeneic human CNS stem cells into the frontal lobe of human patients with early onset Pelizaeus–Merzbacher disease, a congenital dysmyelinating disease, indicated that the procedure was safe and well tolerated, and also resulted in myelination (109). These results are promising and require further investigation to understand mechanism of action. 2P microscopy is a well-suited technique to monitor dynamic interactions between these therapeutic cells and damaged axons in animal models.

**Figure 3:**
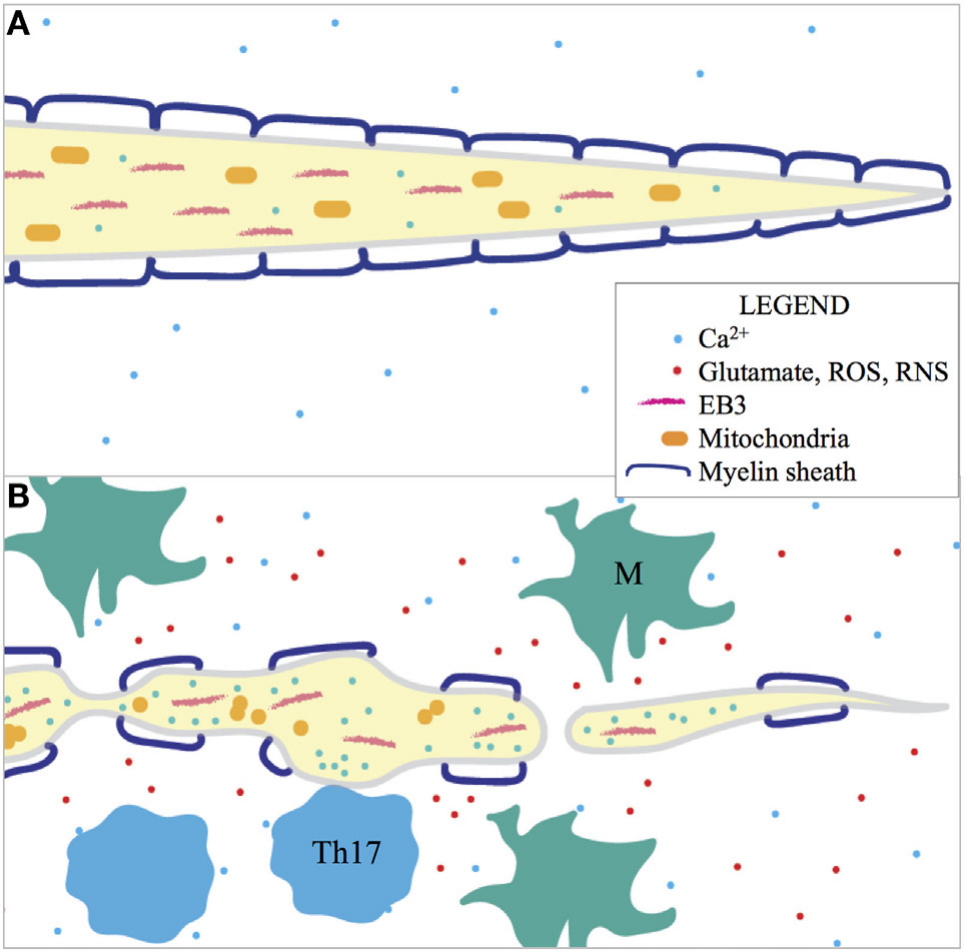
**2P imaging of (A) healthy and (B) degenerating axons in preclinical MS models has shown that many factors are involved in axonopathy and many features can be assayed as indicators of axonal degeneration**. Activated macrophages and microglia (M) release glutamate, ROS, and RNS in the pathogenic environment and these factors contribute to morphological degeneration of the axon (FAD), disorientation of EB3 (indicative of microtubule-based transport), dysmorphia of mitochondria, and increased intracellular [Ca^2+^]. Intracellular [Ca^2+^] is also locally increased during Th17 cell contact in EAE. Organelles, such as mitochondria, are absent in the distal parts of damaged axons. Thus, inflammation is implicated in axonopathy in preclinical MS models.

Electron microscopy and immunohistochemistry implied that syngeneic murine NPCs transplanted into JHMV-infected mice remyelinated axons, and 2P imaging in Thy1-YFP mice confirmed this initial observation (112). Transplanted GFP–NPCs preferentially localized to axons with increasing FAD; engraftment of PLP–GFP–NPCs confirmed that NPCs mature into myelin-producing oligodendrocytes and remyelinated axons (53). Immunostaining of transverse spinal cord sections of PLP–GFP–NPC transplanted JHMV-infected Thy1-YFP mice for myelin basic protein (MBP), another component of myelin, showed that these cells were indeed producing myelin. 2P microscopy allowed for live visualization of spatial juxtaposition of therapeutic NPCs with axons for the first time. A summary of the cellular and molecular components of axonopathy that have been imaged with 2P microscopy in preclinical MS models can be found in Figure 3.

## Conclusion and Future Perspectives

Our perspective is that 2P microscopy is currently the highest resolution methodology for live imaging normal and diseased CNS tissue. A number of advancements make this technology accessible for imaging axonopathy, such as the development of CNS imaging setups, transgenic animals for visualization of axons in the CNS, and various chemicals and dyes to visualize other structures. Thus far, 2P microscopy has facilitated significant gains in understanding axonal degeneration in murine MS models. EAE has been known to be Th17 dependent for some time; with the use of 2P and transgenic Ca^2+^ reporter mice, a unique and novel connection has been made between Th17 cells, FAD, and irreversible neuronal damage. Similarly, while morphological damage to axons has been detected with stains for neurofilament, such as SMI31, SMI32, and Bielschowsky silver impregnation, discovery of the temporal progression in morphological damage was made possible by time-lapse 2P imaging.

Despite this significant progress, whether demyelination or axonal damage occurs first has not been sufficiently answered. 2P data show that structural degeneration, mitochondrial impairment, and ion concentration imbalance may start to occur before demyelination. However, some of these axonal pathologies are products of inflammation, and demyelination is too. But FDA-approved disease-modifying therapies and corticosteroids, which are believed to reduce neuroinflammation, are not believed to prevent onset of progressive disease. Therefore, it may be possible that alternate mechanisms are causing axonal damage, or current therapeutics do not sufficiently decrease neuroinflammation. While the data increasingly suggests the verity of the inside-out model and the possibility that axon damage and demyelination are independent events, their cause may be the same. Continued study of these phenomena is necessary to develop potential therapies.

More research is required to answer the following questions about axonal damage in MS: does axonopathy or demyelination occur first, is a single-target therapeutic sufficient for alleviation of axonopathy, can NSCs/NPCs provide long-term treatment for demyelinating disease? 2P imaging has provided significant gains in moving closer to answering these questions. Thus far, the relationships between ROS, RNS, glutamate, inflammation, and axonal pathologies, such as morphologic damage, transport deficits, mitochondrial deficiencies, and [Ca^2+^] excitotoxicity, have been investigated using 2P. Important areas of study in the future will be mechanism of demyelination onset in viral models, cause and effect of glutamate excitotoxicity, and loss of trophic support by glia and downstream neurons. These are a few of many. Ultimately, by better understanding the complex relationships occurring in neuroinflammatory demyelination, we may stumble upon a single point of convalescence. One of the challenges of developing therapeutics for MS is a lack of understanding about pathogenesis. Together, preclinical murine models and 2P microscopy provide a methodology to understand onset of demyelination and axonopathy. Thus, 2P microscopy may facilitate in gaining a mechanistic understanding of axonal damage in order to develop therapeutics for progressive MS.

## Author Contributions

All authors listed have made substantial, direct, and intellectual contribution to the work, and approved it for publication.

## Conflict of Interest Statement

The authors declare that the research was conducted in the absence of any commercial or financial relationships that could be construed as a potential conflict of interest.

## Abbreviations

2d2, MOG peptide 35–55 TCR transgenic; APP, amyloid precursor protein; ATP, adenosine triphosphate; CerTN-L15, troponin C expressing Ca^2+^ sensing transgenic animal; CFP, cyan fluorescent protein; EAE, experimental autoimmune/allergic encephalomyelitis; EB3, end-binding protein 3; FAD, focal axonal degeneration; FRET, Förster resonance energy transfer; GECI, genetically encoded Ca^2+^ indicator; GFP, green fluorescent protein; JHMV, John Howard Mueller strain of murine hepatitis virus; LFB, Luxol fast blue; MHC, major histocompatibility complex; MOG, myelin oligodendrocyte glycoprotein; MRI, magnetic resonance imaging; MRS, magnetic resonance spectroscopy; MS, multiple sclerosis; NAWM, normal appearing white matter; NPC, neural precursor cell; OT-2, ovalbumin peptide 323–339 TCR transgenic-binding MHC Class II; PLP, proteolipid protein (promoter for mature oligodendrocytes); RAG, recombination activating-gene; RFP, red fluorescent protein; RNS, reactive nitrogen species; ROS, reactive oxygen species; SMI31, Sternberger monoclonal incorporated monoclonal antibody 31; SMI32, Sternberger monoclonal incorporated monoclonal antibody 32; TCR, T-cell receptor; Thy1, thymocyte antigen 1 (promoter for neuron subset); TMEV, Theiler’s murine encephalomyelitis virus; TN-XXL, troponin C expressing Ca^2+^ sensing transgenic animal; XFP, “X” fluorescent protein (any spectral variant of GFP); YFP, yellow fluorescent protein.
